# Dimensional label learning contributes to the development of executive functions

**DOI:** 10.1038/s41598-022-14761-2

**Published:** 2022-06-30

**Authors:** Kara Lowery, Bhoomika Nikam, Aaron T. Buss

**Affiliations:** 1 Department of Psychology, University of Tennessee at Knoxville, Knoxville, USA; 2 Department of Educational Psychology and Counseling, University of Tennessee at Knoxville, Knoxville, USA

**Keywords:** Human behaviour, Attention

## Abstract

A key to understanding how the brain develops is to understand how learning can change brain function. One index of learning that takes place in early childhood involves the comprehension and production of labels describing the shape and color features of objects, a process known as dimensional label learning (DLL). DLL requires integrating auditory and visual stimuli to form a system of mappings that link label representations (e.g. “red” and “color”) and visual feature representations (e.g. “red” and the hue red). Children gain expertise with these labels between the ages of 2 and 5 years, and at the same time they begin to demonstrate skills in using labels to guide cognitive function in other domains. For example, one of the hallmark measures of executive function development requires children to use verbally instructed rules to guide attention to visual dimensions. The broader impact of DLL, however, has not yet been explored. Here, we examine how the neural processes associated with the comprehension and production of labels for visual features predicts later performance on executive function tasks. Specifically, we show that left frontal cortex is activated during comprehension and production tasks at 33 months of age. Moreover, we find that neural activation in this region during label production at 33 months is associated with dimensional attention, but not spatial selective attention, at 45 months. These results shed new light on the role of label learning in developmental changes in brain and behavior. Moreover, these data suggest that dimensional label learning generalizes beyond the learned information to influence other aspects of cognition. We anticipate that these results may serve as a starting point for future work to implement label training as an intervention to influence later cognition.

## Introduction

Early childhood is a time of rapid and profound development of executive function (EF) skills. These skills are associated with a wide range of developmental outcomes including academic achievement^1–12^. Identifying early neural markers that can predict later EF development can inform our understanding of the mechanisms involved in EF development and the ways in which EF is related to other aspects of cognitive functioning. Until recently, however, examination of the neural mechanisms associated with the early emergence of EF has remained elusive. This fact is primarily due to limitations in technology available to measure neural function during early childhood but is also due to limitations in theories of neurocognitive development. Typically, EF development is explained via maturational processes. In these theories, the learning or experience-driven processes that change brain function are not specified^13,14^. Due to the abstract and general nature of EF processes, it is challenging to link these processes to more specific learning mechanisms that are involved in the emergence of EF. Without identifying which more fundamental processes give rise to EF, it can be challenging to know where to look for the early precursors of EF. In this project, we focus on a specific aspect of EF: dimensional attention. Dimensional attention refers to the ability to focus processing on a specific aspect of stimuli, such as shape or color. This includes abilities such as selective and flexible attention^15^.

A recent neurocomputational theory, dynamic field theory, has demonstrated that learning labels for visual features and dimensions can explain the development of task-general attentional skills across a wide range of contexts, including rule-use, categorization, and priming^15^. In this framework, learning labels for visual features builds associations between labels and visual representations. These associations can then be used to prioritize processing of information based on the represented task goals. Thus, it is proposed that label learning cultivates neural mechanisms that can be used to think in flexible or goal-directed ways based on how labels are used to enhance processing of associated feature information. A key prediction of this model is that comprehension and production of dimensional labels should elicit neural activation that is meaningfully related to the development of dimensional attention.

This perspective follows in the spirit of a recent reconceptualization of EF around the skills that children use to pursue goals in specific contexts^16–18^. That is, EF is typically conceptualized as having structural components corresponding to inhibition, working memory, and cognitive flexibility^19^. This new reconceptualization instead proposes that EF arises from a collection of factors including the child’s cognitive skills, values, and motivations as well as the context or environment in which children are pursuing goals. Thus, rather than framing performance on EF tasks such as the dimensional change card sort (DCCS) task around structural components of EF^13,20,21^, this model couches performance on this task around the use of dimensional labels to enhance processing of visual dimensions. The success or failure of children on this task and associated tasks is then a function of the configuration of stimuli and history of decisions in the task or experiences preceding the task^15,22–25^.

In this project, we explored the neural mechanisms involved in the early understanding of labels for visual features and dimensions. Assessments of dimensional label learning typically involve simple measures of comprehension in which children select a target object from an array (“Which one is red?”) and production in which children are prompted to produce a color label (“What color is this?”). The process of learning labels for visual features is a well-defined problem space. Children learn direct associations between visual features such as the hue of red and the label ‘red’. Children also form direct associations between labels and other labels. For example, children can reliably provide a color label (even if not the correct label) when asked, “what color is this one?”. In this way, children understand that certain labels are related to one another. As children form an integrated set of associations of labels with features and labels with other labels, they can produce the correct label when asked “what color is this one?”^26^. Although these examples focus on the dimension of color, similar patterns of learning have also been observed with the dimension of shape^27^. In general, children are deemed to have acquired their dimensional labels by around 30 months^26,27^.

Dimensional label learning has also been linked to an understanding of dimensionality. That is, as children learn the network of label-label-feature associations, they develop an understanding of visual dimensions that gives rise to the ability to attend to those dimensions^26^. For example, in assessments of dimensional label learning, a matching task is administered to probe children’s dimensional attention^26^. In this task, children are shown a pair of reference objects that are the same color, but different shapes. Children are then prompted to select from an array another object that is the same as the first pair of objects. If children can appreciate that the original pair of objects is not just the same in an arbitrary way, but their sameness is of a dimensional nature, then children can reliably identify another object that matches along the color dimension.

Dimensional labels become more significant later in development as they are not just used for an understanding of dimensionality but are used in tasks that measure the early emergence of EF. For example, the DCCS task tests children’s cognitive flexibility using verbally administered rules. Children are given an initial set of rules to sort cards by shape or color and then to switch and sort cards by the other dimension. Such measures of performance are often used to gauge the developmental status of EF. This task reveals a qualitative shift in performance: the majority of 3-year-olds typically fail to switch rules but have little difficulty switching by age 4^28^. Moreover, performance on the DCCS is impaired in populations with developmental language disorder^29^, attention deficit hyperactivity disorder^30^, and autism^31^. Thus, the DCCS task provides an important index of dimensional attention control during early development. The relationship between dimensional label learning and the DCCS task, however, has not been previously explored. Previous research has, however, demonstrated a role of labeling and dimensional understanding on DCCS performance. For example, having children label the relevant feature of the test card prior to sorting improves their ability to switch rules^32,33^. Further, training children on dimensional understanding prior to performing the DCCS by having them separate and aggregate the dimensions of objects can also enhance switching^34^. Moreover, performance on the DCCS is associated with performance on measures of attentional priming and categorization that do not involve explicit instructions to attend to dimensions^15,35^. Thus, the skills required for the DCCS generalize to other contexts in which attention is implicit.

**Figure 1:**
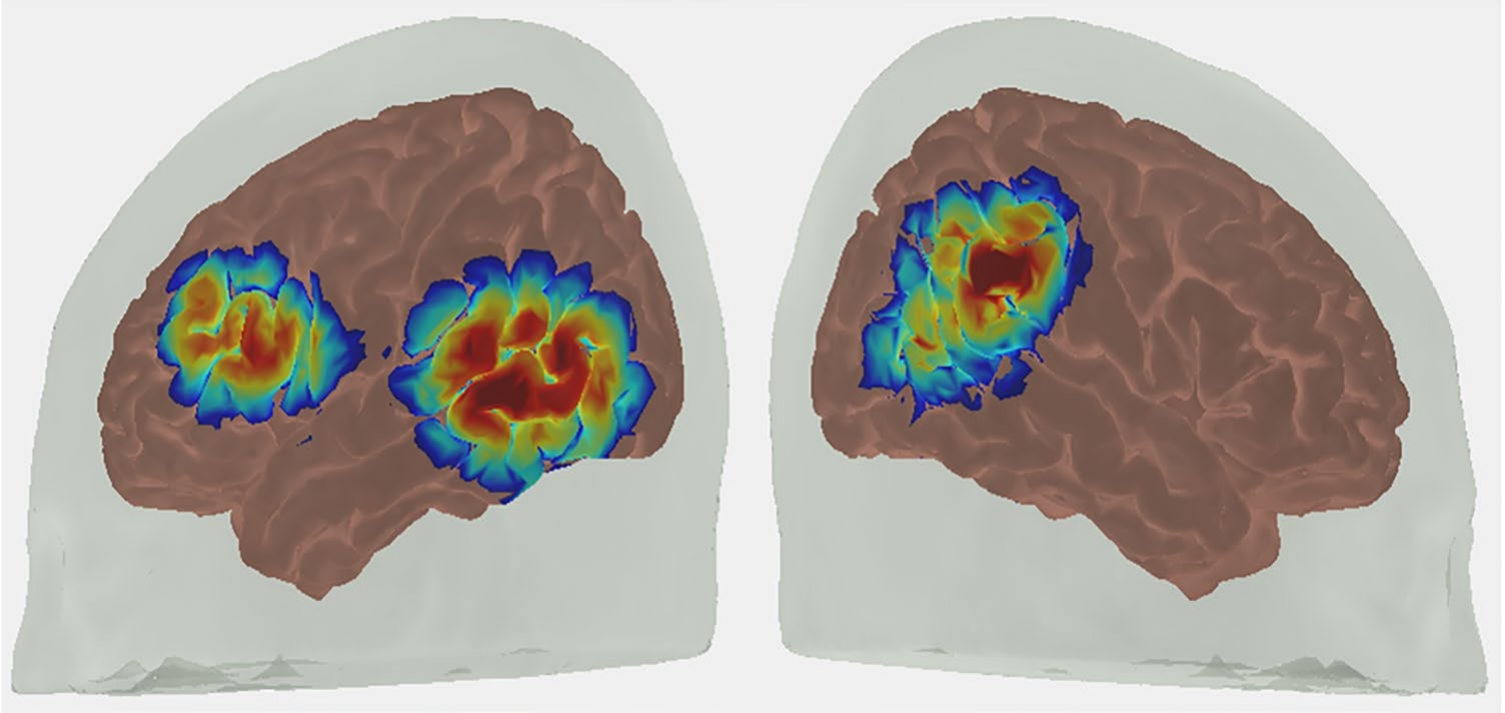
Sensitivity profile of fNIRS probe. Sources and detectors were placed over left frontal and bilateral temporal-parietal regions.

**Figure 2:**
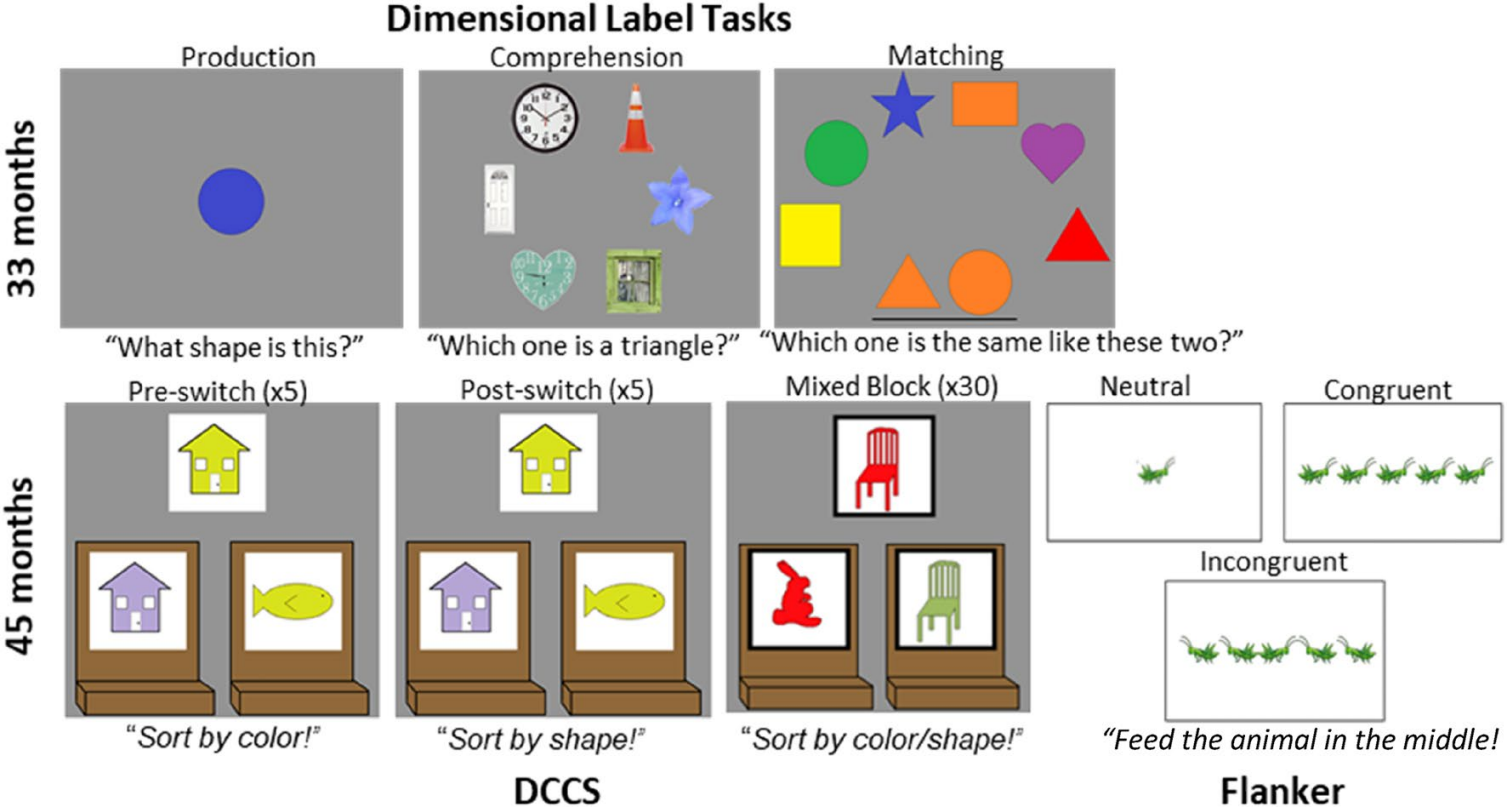
Tasks completed at 33 and 45 months. (33 months) Children completed production, comprehension, and matching tasks for colors, shapes, and embedded shapes. (45 months) Children completed DCCS and Flanker tasks, as well as production and comprehension tasks for colors and shapes.

In the current study, we made two advancements. First, we used functional near-infrared spectroscopy (fNIRS) to measure the neural mechanisms involved in the comprehension and production of dimensional labels and dimensional attention at 33 months of age. We recorded neural data from left frontal cortex and bilateral parietal-temporal cortices (Fig. 1) which are regions that have previously been identified in the development of successful switching in the DCCS task^25^. Comprehension and production tasks were administered with canonical colors (red, orange, yellow, green, blue, purple) and shapes (circle, square, triangle, star, rectangle, heart), as well as embedded shapes (Fig. 2). Stimuli in the embedded shapes tasks were images of real-world objects that had a canonical shape (e.g., clock for circle, a traffic cone for triangle). With these factors, we compared activation as a function of task (comprehension vs production) as well as dimension (color vs shape) and stimulus complexity (canonical shapes vs embedded shapes). Lastly, we administered a dimensional matching task to measure cognitive skills and neural processes associated with dimensional attention at 33 months of age. In this case, we compared neural activation as a function of stimulus dimension (shape vs color) and stimulus complexity (canonical shapes vs embedded shapes). In addition, we identified neural activation associated with the dimensional attention demands of the matching task by comparing against the comprehension task. Both of the tasks require children to select a target object from an array, but dimensional attention is required in the matching task.

We then tested the longitudinal relationship between these early measures of neural activation and later developing dimensional attention. Specifically, we administered the DCCS task and the flanker task at 45 months of age. The DCCS is typically administered in two blocks (pre-switch and post-switch) with behavior coded as pass/fail based on whether the post-switch trials are performed correctly. This method, however, is not conducive to examining individual difference since children are categorized into two groups. To obtain a continuous measure of individual differences in dimensional attention skills, we administered a mixed block after the post-switch phase in which the stimuli were switched to different shapes and colors than those used during the pre- and post-switch phases. During this phase of the task, the relevant dimension was randomized trial to trial with 1/3 of the trials instructed on the dimension that was relevant for the pre-switch phase and 2/3 of the trials instructed on the dimension that was relevant for the post-switch phase. We then defined performance as the total percent correct across all trials. This method allowed us to obtain a measure of each child’s dimensional attention skills in as individuated a manner as possible^15,36^. All children sorted correctly on at least 4 out of 5 trials during the pre-switch phase.

The flanker task was administered as a control task in which dimensional attention is not thought to be required for successful performance. The flanker task is primarily a measure of spatial selective attention, whereas the DCCS requires dimensional attention. For this reason, the flanker task was used as a control to ensure that any relationship between the DCCS and early dimensional label learning is due to dimensional attention. Further, research has shown that performance on the DCCS and flanker tasks are not associated with one another suggesting that they engage distinct cognitive processes^37^. In our version of the flanker task, we used animal stimuli modeled after tasks previously used in the literature^38^. Children were instructed to press a left and right button to feed the animal presented at the middle of the screen by pressing the button that matched the direction the animal was facing. On neutral trials, only the target stimulus was shown on the screen. On congruent trials, the target object was flanked by four task-irrelevant animals (two on either side) that were facing the same direction as the target stimulus. On incongruent trials, the flanking items were facing the opposite direction as the target stimulus. In this task, we can analyze basic response selection processes such as reaction-time and accuracy. Typically, participants are slower and more error prone on incongruent trials compared to neutral and congruent^39^. This task has not been previously used with this young of a population but will provide an index of EF that is expected to be distinct from the DCCS and dimensional label learning.

Our primary hypothesis is that learning labels for visual dimensions creates associations between labels and visual features that are used to guide dimensional attention. To test this hypothesis, we will first identify neural activation associated with the comprehension and production of dimensional labels at 33 months of age. We will then use parametric Pearson correlations to examine how neural activation is associated with subsequent measures of dimensional and spatial selective attention at 45 months of age. If the association between early dimensional label learning and measures of attention is specific to dimensional attention, then neural activation at 33 months would selectively predict performance on the DCCS task but not the flanker task.

## Results

### Behavioral results at 33 months

We compared performance on the comprehension and production tasks for canonical shapes and colors using a 2 × 2 repeated measures ANOVA. We observed a main effect of Dimension, *F*(1,24) = 5.716, *p* = 0.025, ηp^2^ = 0.192. Children performed better with colors (*M* = 0.752, SD = 0.30) than shapes (*M* = 0.660, SD = 0.25). We also observed an interaction between Task and Dimension, *F*(1,24) = 4.255, *p* = 0.05, ηp^2^ = 0.151. Children did not differ on comprehension performance between dimensions (color *M* = 0.712, SD = 0.32; shape *M* = 0.684, SD = 0.25), *t*(24) = 0.562, *p* = 0.579, but were better at color production (*M* = 0.792, SD = 0.28) than shape production (*M* = 0.636, SD = 0.25), *t*(24) = 3.183, *p* = 0.004. We examined the impact of stimulus complexity on dimensional label comprehension and production by comparing performance between canonical shapes and embedded shapes. In this analysis, we observed a main effect of Complexity, *F*(1,24) = 18.782, *p* < 0.001, ηp^2^ = 0.439. Performance with canonical shapes (*M* = 0.66, SD = 0.25) was better than performance with embedded shapes (*M* = 0.53, SD = 0.26).

We next examined dimensional attention performance at 33 months by comparing performance on the matching tasks across the three dimensions using two paired-sample *t*-tests (two-tailed). First, we examined the effect of Dimension. Performance between canonical shapes (*M* = 0.42, SD = 0.37) and colors (*M* = 0.45, SD = 0.37) did not differ, *t*(24) = 0.291, *p* = 0.773. When examining the effect of Complexity, we found that performance with embedded shapes (*M* = 0.27, SD = 0.22) was significantly worse than with canonical shapes, *t*(24) = 2.265, *p* = 0.033. Lastly, we examined the associations between dimensional label understanding and dimensional attention at 33 months. Total production percent correct (r = 0.437, p = 0.09) and total comprehension percent correct (r = 0.438, p = 0.014) were both associated with total matching percent correct.

### Behavioral results at 45 months

Twelve out of the twenty-five children who completed the DCCS failed to switch rules. This rate of switching is typical for this age^20^. The average proportion correct during the mixed block was 0.50. A paired-samples *t-*test (two-tailed) showed that children’s mixed block performance did not differ depending on whether they passed (*M* = 0.51) or failed (*M* = 0.49) during the post-switch phase, *t*(11) = − 0.21, *p* = 0.837. For brain-behavior analyses described below, we calculated a total score as the percent correct across all test trials (pre-switch, post-switch, and mixed block).

For the flanker task, we conducted two repeated-measures ANOVAs to examine the effects of Trial Type (neutral, congruent, and incongruent) on accuracy and reaction time. There was a main effect of Trial Type for accuracy, *F*(2,48) = 21.630, *p* < 0.001, ηp^2^ = 0.474 such that children performed worse on incongruent trials (*M* = 0.61, SD = 0.21) than neutral (*M* = 0.89, SD = 0.18, *p* < 0.001) and congruent trials (*M* = 0.83, SD = 0.18, *p* = 0.001). Neutral and congruent trials did not differ, *p* = 0.22. We also found a main effect of Trial Type for reaction time, *F*(2,48) = 22.385, *p* < 0.001, ηp^2^ = 0.483. Children were faster on neutral trials (*M* = 2893 ms, SD = 970 ms) than both congruent (*M* = 3413 ms, SD = 1382 ms, *p* = 0.005) and incongruent trials (*M* = 4348 ms, SD = 1760 ms, *p* < 0.001), and faster on congruent trials than incongruent trials (*p* = 0.002). For brain-behavior analyses described below, we calculated three scores that were used from the flanker task: percent correct on incongruent trials, the difference in accuracy between congruent and incongruent trials, and the difference in accuracy between neutral and incongruent trials. DCCS task performance was not related to any aspect of flanker task performance (all *p* > 0.131).

### Behavioral associations between 33 and 45 months

We next examined associations between behavioral tasks administered at 33 and 45 months (see Table 1 for statistical results). DCCS performance was not associated with performance on any of the tasks administered at 33 months. In the flanker task, percent correct on neutral trials was associated with percent correct on color production, shape production, color comprehension, and color matching. Percent correct on congruent trials was associated with percent correct on color shape production, color comprehension, shape comprehension, and color matching. Percent correct on incongruent trials was not associated with percent correct on any of the tasks administered at 33 months. The flanker effect relative to neutral trials was associated with percent correct on color production and shape matching.

**Table 1:** Correlations between dimensional label tasks and EF tasks (flanker and DCCS).

	Production			Comprehension			Match		
	Color	Shape	Emb	Color	Shape	Emb	Color	Shape	Emb
DCCS	r = − 0.117 p = 0.577	r = − 0.052 p = 0.805	r = − 0.058 p = 0.782	r = 0.064 p = 0.760	r = − 0.129 p = 0.539	r = 0.073 p = 0.730	r = − 0.132 p = 0.530	r = − 0.140 p = 0.503	r = − 0.194 p = 0.353
Flanker: N	**r = 0.559** **p = 0.004**	**r = 0.515** **p = 0.008**	r = 0.310 p = 0.132	**r = 0.567** **p = 0.003**	r = 0.391 p = 0.053	r = 0.324 p = 0.114	**r = 0.479** **p = 0.015**	r = 0.320 p = 0.118	r = 0.131 p = 0.531
Flanker: C	r = 0.348 p = 0.088	**r = 0.441** **p = 0.028**	r = 0.251 p = 0.227	**r = 0.427** **p = 0.033**	**r = 0.593** **p = 0.002**	r = 0.339 p = 0.098	**r = 0.442** **p = 0.027**	r = 0.352 p = 0.084	r = 0.248 p = 0.233
Flanker: I	r = − 0.049 p = 0.817	r = 0.077 p = 0.715	r = 0.107 p = 0.610	r = 0.198 p = 0.342	r = 0.160 p = 0.443	r = 0.062 p = 0.770	r = 0.121 p = 0.566	r = − 0.178 p = 0.396	r = 0.187 p = 0.372
Flanker: C–I	r = 0.271 p = 0.190	r = 0.232 p = 0.265	r = 0.084 p = 0.691	r = 0.126 p = 0.549	r = 0.268 p = 0.195	r = 0.175 p = 0.404	r = 0.199 p = 0.340	r = 0.368 p = 0.071	r = 0.019 p = 0.930
Flanker: N–I	**r = 0.469** **p = 0.018**	r = 0.322 p = 0.116	r = 0.140 p = 0.505	r = 0.249 p = 0.231	r = 0.154 p = 0.462	r = 0.188 p = 0.368	r = 0.256 p = 0.216	**r = 0.397** **p = 0.050**	r = − 0.068 p = 0.748

Significant correlations are highlighted in bold. *N* Neutral, *I* Incongruent, *C* Congruent.

### Neural results at 33 months

**Figure 3:**
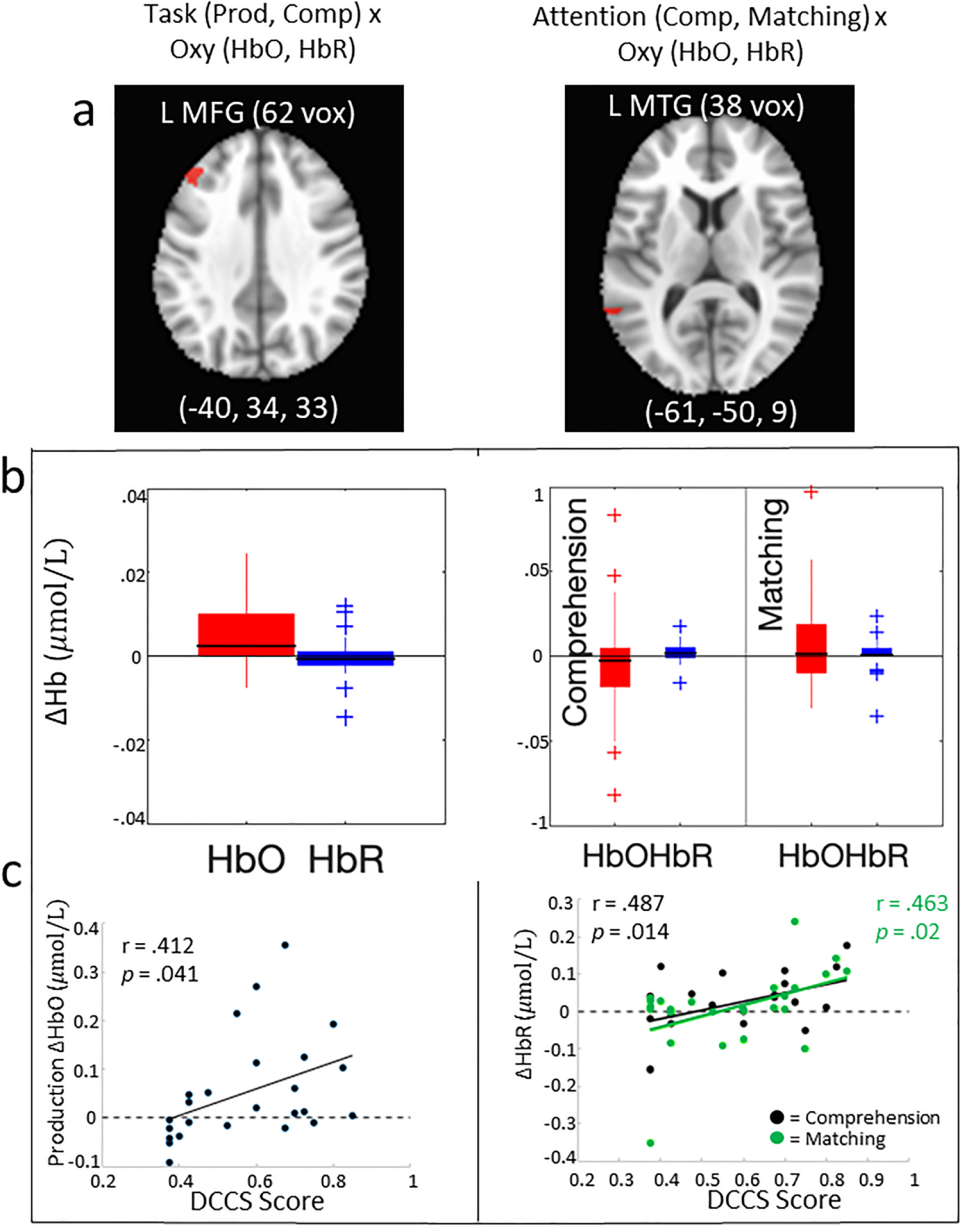
DCCS scores at 45 months were associated with DL activation at 33 months. (**a**) Location of clusters displaying activation in L MFG (middle frontal gyrus) and L MTG (middle temporal gyrus) (**b**) Boxplots of Hb values from clusters. Bars show the range from 25 to 75 percentiles of data. The median is marked by black line. Whiskers mark the range of data not considered outliers and outliers are indicated by + makers. (**c**) Hb concentration during the DL tasks at 33 months was associated with DCCS performance at 45 months old.

First, we examined neural activation as a function of labeling task. That is, we compared comprehension against production and included all stimulus types within these tasks (Fig. 3a,b). We observed a cluster of activation in left middle frontal gyrus (MFG) which showed an effect of Hb, *F*(1,24) = 6.379, *p* = 0.018, η^2^ = 0.08. This region of the brain showed significantly higher HbO (*M* = 0.043) compared to HbR (*M* = − 0.011) suggesting that this region of cortex was engaged during both the comprehension and production tasks. Next, we examined neural activation that was unique to dimensional attention by comparing activation during the matching task and the comprehension task. This comparison was chosen because the comprehension task has similar task requirements as the matching task (i.e., find a target among an array of items) but does not require dimensional attention to locate the target. This analysis revealed a cluster in left middle temporal gyrus (MTG) that showed an interaction between Hb and Task, *F*(1,24) = 5.548, *p* = 0.027, η^2^ = 0.02. Follow-up analyses showed that the difference between HbO and HbR was higher during the matching task (*M* = 0.08) than during the comprehension task (*M* = − 0.07, *p* = 0.027). Thus, this region was more strongly activated when dimensional attention was required.

Next, we examined differences between dimensions by comparing activation between the shape and color dimensions. That is, we compared shape trials against color trials and included both comprehension and production conditions. This analysis did not yield any significant clusters. Lastly, we examined the effect of stimulus complexity by comparing activation between the shape and embedded shape tasks. Again, we compared canonical shape trials against embedded shape trials and included both comprehension and production conditions. This analysis also did not reveal any significant clusters.

**Figure 4:**
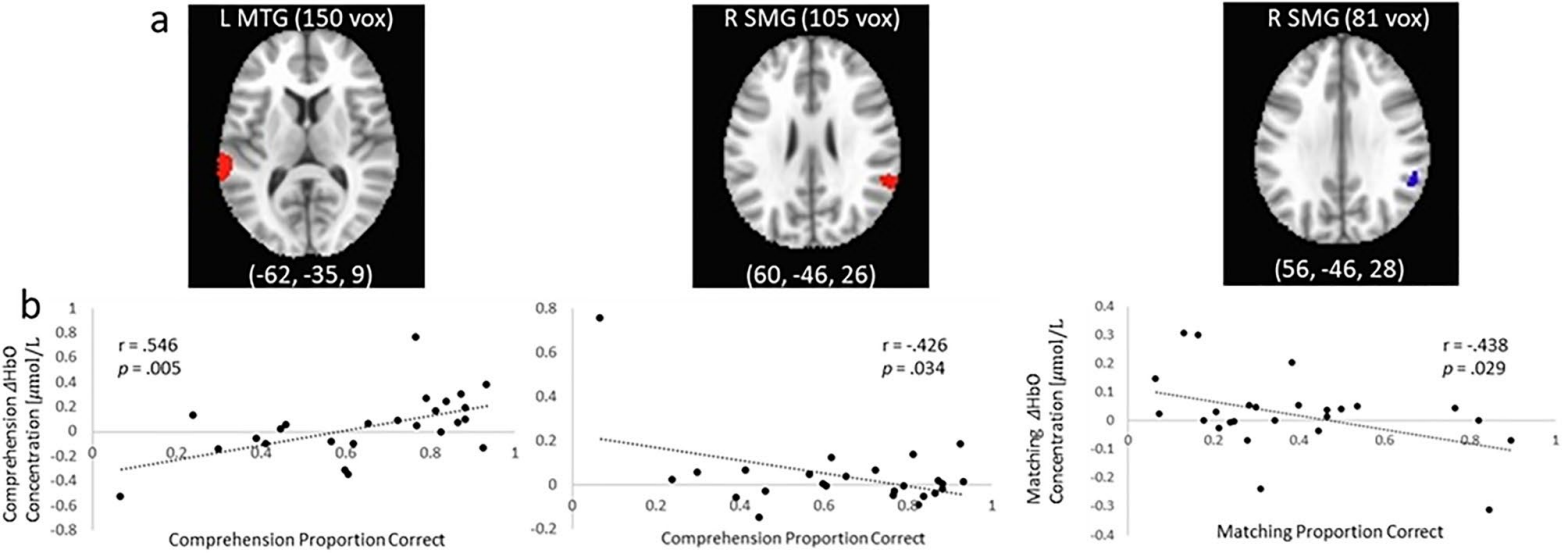
Comprehension and matching activation and performance at 33 months were associated. (**a**) Location of clusters displaying activation in L MTG (middle temporal gyrus) and R SMG (supramarginal gyrus) (**b**) Comprehension and matching performance were associated with task activation.

We also examined whether label task performance was associated with activation during the label tasks (Fig. 4a,b). HbO in left MTG during dimensional label comprehension was positively associated with comprehension performance, such that children who had higher performance also exhibited higher activation (*r* = 0.546, *p* = 0.005). We also found a negative relationship between comprehension activation and performance in right supramarginal gyrus (SMG), indicating that children with higher performance displayed lower activation in this region (*r* = −0.426, *p* = 0.043). There was a similar negative association in right SMG between matching performance and activation (*r* = − 0.438, *p* = 0.029). There were no task-activation associations for production. All correlations were conducted using two-tailed tests.

### Predicting behavior at 45 months

Our primary question is whether the neural processes involved in the comprehension and production of dimensional labels is predictive of later development of dimensional attention. We used the clusters identified above to examine whether activation within these regions predicted performance on the DCCS and flanker tasks at 45 months (Fig. 3c). For the DCCS task, we used total percent correct. For the flanker task, we examined multiple measures of performance: accuracy on incongruent, flanker effect relative to congruent trials (difference between accuracy on congruent and incongruent), and flanker effect relative to neutral trials (difference between accuracy on neutral and incongruent). Because we are making predictions that one set of behavioral measures will not be associated with activation, we also present the Bayes factor (BF) for tests that are non-significant.

HbO in left MFG during label production at 33 months of age was significantly positively associated with DCCS performance at 45 months of age (*r* = 0.412, *p* = 0.041). One concern is that this association may be driven by outliers. To examine the robustness of this association we removed one participant whose activation was more than 2.5 *SD* above the average activation. The correlation was still significant (*r* = 0.417, *p* = 0.043). We also removed one additional participant whose activation was more than 2 *SD* above the average activation and the correlation was still significant (*r* = 0.473, *p* = 0.023). HbO in this cluster during label production was not associated with flanker performance (incongruent: *r* = − 0.172, *p* = 0.411, *BF* = 4.646; congruent–incongruent: *r* = 0.056, *p* = 0.790, *BF* = 6.276; neutral–incongruent: *r* = 0.226, *p* = 0.276, *BF* = 3.607). HbR in this region during label production at 33 months of age was not associated with any behavioral measure (DCCS: *r* = 0.105, *p* = 0.616, *BF* = 5.738; incongruent: *r* = 0.139, *p* = 0.506, *BF* = 5.219; congruent–incongruent: *r* = 0.029, *p* = 0.891, *BF* = 6.440; neutral–incongruent: *r* = − 0.130, *p* = 0.534, *BF* = 5.365).

HbO in this region during the comprehension task was not associated with any behavioral measures (DCCS: *r* = − 0.041, *p* = 0.847, *BF* = 6.381; incongruent: *r* = − 0.244, *p* = 0.240, *BF* = 3.273; congruent–incongruent: *r* = 0.195, *p* = 0.350, *BF* = 4.214; neutral–incongruent: *r* = 0.123, *p* = 0.558, *BF* = 5.479). HbR in this region during the comprehension task was also not associated with any behavioral measures (DCCS: *r* = 0.015, *p* = 0.942, *BF* = 6.438; incongruent: *r* = − 0.230, *p* = 0.270, *BF* = 3.549; congruent–incongruent: *r* = 0.370, *p* = 0.069, *BF* = 1.256; neutral–incongruent: *r* = 0.312, *p* = 0.129, *BF* = 2.063).

Within the cluster of activation from the comparison between comprehension and matching in left middle temporal gyrus, DCCS performance was significantly positively correlated with HbR for both comprehension and matching tasks (*r* = 0.487, *p* = 0.014 and *r* = 0.463, *p* = 0.02, respectively). This result suggests that better DCCS performance was associated with weaker activation in this cluster during the comprehension and matching tasks. Flanker performance was not associated with HbR values for comprehension (incongruent: *r* = 0.344, *p* = 0.092, *BF* = 1.590; congruent–incongruent: *r* = − 0.328, *p* = 0.110, *BF* = 1.824; neutral–incongruent: *r* = − 0.036 *p* = 0.865, *BF* = 6.408) nor matching (incongruent: *r* = 0.066, *p* = 0.754, *BF* = 6.192; congruent–incongruent: *r* = − 0.149, *p* = 0.477, *BF* = 5.058; neutral–incongruent: *r* = − 0.102, *p* = 0.629, *BF* = 5.787). Behavioral performance was not associated with HbO during comprehension (DCCS: *r* = − 0.270, *p* = 0.191, *BF* = 2.718; incongruent: *r* = − 0.059, *p* = 0.780, *BF* = 6.253; congruent–incongruent: *r* = 0.080, *p* = 0.705, *BF* = 6.055; neutral–incongruent: *r* = 0.145, *p* = 0.491, *BF* = 5.133) nor matching (DCCS: *r* = − 0.152, *p* = 0.468, *BF* = 5.003; incongruent: *r* = − 0.038, *p* = 0.857, *BF* = 6.396; congruent–incongruent: *r* = 0.204, *p* = 0.329, *BF* = 4.049; neutral–incongruent: *r* = 0.042, *p* = 0.843, *BF* = 6.374) tasks.

## Discussion

The present study examined early neural markers of later executive function (EF) development. Measures of neurocognitive function during early childhood are rare and typically limited to passive tasks. fNIRS, however, can provide measures of neural function during tasks in which children are actively engaging cognitive functions. Here, we focused specifically on one aspect of EF, dimensional attention. Based on the dynamic neural field model of dimensional attention^15^, we predicted that neural activation during the comprehension and production of dimensional labels would be meaningfully related to later performance on the DCCS task.

We provided the first examination of the neural mechanisms underlying dimensional label comprehension and production. Our results showed that left frontal cortex is engaged when children perform the simple comprehension and production of dimensional labels. We also examined dimensional attention at 33 months of age and found that left temporal cortex is engaged when demands are placed on dimensional attention. As predicted by the dynamic neural field model, neural responses during dimensional label production at 33 months of age were predictive of performance on the DCCS, but not the flanker task. This finding supports the idea that early dimensional label learning influences later dimensional attention, one aspect of EF. This is particularly noteworthy because of the wide generalization of the effect of label learning. That is, the DCCS is typically characterized as a test of cognitive flexibility and rule-use^20,40^ which are not task demands involved in the dimensional label production task. Nevertheless, the neural processes involved in the production of dimensional labels was predictive of future performance on the DCCS task. Bayes factors on the correlations between flanker performance and neural activation during the DL tasks ranged from anecdotal to moderate evidence for the null hypothesis. Thus, future work will be needed to replicate these results with larger samples and perhaps a wider array of control tasks to better understand the relationship between early neural measures and later measures of EF.

It is noteworthy that activation during dimensional label production, but not comprehension, was associated with later performance on the DCCS task. As discussed by Sandhofer and Smith^26^, dimensional label production requires integrating multiple types of associations. That is, the dimensional label is associated with a subset of feature labels, and the feature labels are associated with specific feature values. Thus, when asked, for example, “What color is this?”, children must use the label “color” to prime the set of labels relevant for that dimension. These labels can then be used to boost neural representation of the relevant visual dimension to select the appropriate feature for processing. Once the visual feature is selected, the associated feature label can be selected for the response. In contrast, the comprehension task provides children with the feature label, and simple feature-label associations can be used to select the appropriate target object. Thus, the production task uniquely involves visual attention which may be the reason that neural activation during this task was associated with later DCCS performance.

These data support a new interpretation of the mechanistic basis of EF development. Typically, EF is described in terms of components such as inhibition, working memory, and switching. The development of these components is ascribed to maturation of frontal cortex, and training or intervention studies typically focus on directly training these components^41^. Aside from training these components as one would work out a muscle, there is no room for learning or experience-specific processes to create developmental changes in EF. Our results implicate a specific learning process, associating visual features with labels, as a driver of changes in dimensional attention. Based on the previous work using the DNF model^15,22,25^, forming associations between visual features and labels provides a mechanism by which label representations can influence object representations and guide goal-directed behavior. The current study is the first to directly demonstrate that the processes of dimensional label learning influence the neurocognitive dynamics of EF development.

We also observed activation in left middle temporal cortex related to the dimensional attention demands of the matching task. Previous research has implicated this aspect of temporal cortex to be important for aspects of cognition such as color naming and semantic memory—the binding of conceptual representations to long-term memory^42,43^. Therefore, it is not surprising that we would see activation in these regions during these dimensional label learning tasks. What was perhaps surprising was that HbR in this region was positively associated with future DCCS performance. This finding suggests that development may involve not only an increase in frontal cortex activation, but also a decrease in posterior cortical activation on the dimensional label tasks.

A number of other behavioral observations were also made in this study. At 33-months of age, we found that children had lower performance with the embedded shape tasks compared to canonical shape tasks, which is consistent with previous findings in the literature that embedded shapes are particularly difficult^27^. This difficulty could be due to the need for selective attention to draw focus to the shape label applicable to the embedded shape and not the associated object label. Mutual exclusivity could also have played a role, in that once children have one label for an object (e.g., a clock), they are resistant to applying another label to it (e.g., circle). We also found that children had better performance in the color tasks than shape tasks. This is not surprising, as previous research shows that color labels are much more frequent in the linguistic input that children receive^44^. Children also completed three different types of dimensional tasks: production, comprehension, and matching. We found that children had poorer performance with matching tasks than with production or comprehension, consistent with previous findings from these tasks^26^. Difficulty on the matching task could also be due to the increased demands on selective dimensional attention.

At 45 months of age we observed that a large portion of children failed to switch rules (11/25) during the post-switch phase of the DCCS task which is typical for this age group^20^. Additionally, we also observed that performance on the mixed block was not related to success on the post-switch phase, suggesting that the mixed block provides a unique measure of attentional flexibility. During the mixed block, the features of the cards are changed to new values which typically improves switching performance^20^. The random intermixing of which dimension is relevant from trial to trial, however, presented task demands that were resolved with different degrees of success across participants. On a flanker task, we found that children had the lowest performance and highest reaction time on incongruent trials, which is consistent with previous findings in the literature with older children and adults^38,39^. Additionally, children were slower on congruent trials compared to neutral trials. This finding suggests that 45-month-olds have difficulty focusing attention on the task relevant item even when distracting stimuli are congruent with the identity of the target stimulus. Very few studies have explored the effect in younger children, especially using directional stimuli^45^. Nonetheless, these results are generally consistent with results from a modified version of the attentional network task (ANT) that has been used to study the flanker effect in children as young as 4 years of age^38^.

We also observed that behavioral measures of DL performance were associated with later measures of flanker performance. Percent correct on neutral and congruent trials were associated with accuracy measures during different production, comprehension and matching tasks administered at 33 months (see Table 1). These associations suggest that basic response selection processes are associated over this time frame. Most interestingly, the measure of the flanker effect relative to neutral trials was associated with color production and shape matching. The pattern showed that children with larger reductions in accuracy on the flanker trials relative to neutral trials at 33 months had higher levels of accuracy on color production and shape matching trials at 45 months. These results suggest that the DL tasks may also involve an aspect of inhibitory control that is also involved in the flanker task. For example, when performing the production task, children may require inhibitory control to suppress other associated labels that are correct for the current target. Neural mechanisms of inhibitory control are often localized to the right frontal cortex which we did not monitor in the current study. Thus, future studies should examine how other cortical regions beyond those studied here may relate between dimensional label learning and different aspects of EF.

It is also important to note the limitations to the current study. While fNIRS offers many advantages to other types of neuroimaging, especially with this age group due to motion artifacts, one limitation is that near-infrared light can only reach surface cortical areas. Therefore, we would be unable to determine whether deeper areas were associated with label learning tasks. Relatedly, we were only able to measure from a couple targeted regions of cortex based on our probe design. Another limitation is that we did not screen for color blindness, so we do not know whether children in our sample had deficient color perception. Furthermore, of the children that started performing the protocols, only about 2/3 completed the study. Thus, there may have been some selection bias in the children that made it through data collection meaning that children who made it into our analyses may be different from typical 33-month-olds. It is important to point out, however, that the 2- to 3-year-old age range is particularly challenging to study in neuroimaging research and the data provided here are the first of its kind with this age group.

## Summary

The current study examined neural markers that are predictive of later dimensional attention development. This is the first study to examine the neural basis of dimensional label learning in toddlers. The current findings showed that neural activation during comprehension and production of dimensional labels during childhood recruits activation of frontal cortex. In addition, activation in these tasks at 33 months predicted performance on the DCCS task (but not the flanker task) one year later. This observation sheds new light on the nature of processes that serve as building blocks for executive function. Based on the neurocomputational framework provided by dynamic field theory, these results suggest that dimensional label learning influences the development of dimensional attention. Future work will investigate whether manipulating dimensional label learning through specific training influences dimensional attention later in development and how these measures of attention in early development relate to later measures of school-readiness.

## Methods

### Experimental procedures

#### Participants

Twenty-five children were included in the final analyses (18 girls; first session: M = 33.4 months, SD = 0.45 s session: M = 45.7, SD = 0.59). We did not conduct any participant screening. An additional 40 children were recruited but were excluded for various reasons. Twelve children failed to finish task protocols in the first session, twelve children refused to begin the tasks at all, two were excluded for parental interference during the session, another because the fNIRS hat was too small for the participant’s head, three due to experimenter error, one due to an unusable digitization of the fNIRS probe, one child’s age had been incorrectly reported in our participant database, and eight children who had successfully completed the session at 33 months did not return for the session at 45 months. Each session took no longer than an hour, and the children were given a toy for participating. At the completion of the second session, families were also given a $10 Amazon gift card. The University of Tennessee, Knoxville Institutional Review Board (IRB) approved the study protocol, and all methods were performed in accordance with guidelines and regulations. Informed consent was obtained from the parents or legal guardians of all participants.

#### Behavioral tasks

At 33-months, children completed production, comprehension, and matching tasks for colors, canonical shapes, and embedded shapes, similar to tasks used previously in the literature^26,27^ on a 27-inch touchscreen enabled monitor. Each of the nine tasks included ten trials (for a total of 90). The color tasks used six different colors (red, orange, yellow, green, blue, and purple), while the shape tasks used six different shapes (circle, square, triangle, rectangle, heart, and star, Fig. 2). These tasks were administered on a PC running E-Prime 2.0 software. For the production tasks, children were shown a single object and asked, “What shape/color is this?”. The experimenter entered the child’s responses into two categories based on whether the child responded correctly or incorrectly. If children stated they did not know, this was coded as an incorrect answer. If children first answered with an irrelevant dimension, the experimenter prompted them by saying, “I know that’s a [child’s response], but do you know what color/shape it is?”. The experimenter only input the child’s response if the child then answered in the relevant dimension or repeated their previous answer. For the comprehension tasks, children were shown an array of six objects that were placed along an imaginary circle and were asked, for example, “Which one is blue”. Children responded by touching one of the six objects on the screen. For the matching tasks, children were shown two objects that were the same along one dimension and were asked, “Do you see how these two are the same?” [pointing to the two reference objects]. After 2000 ms, an array of six other objects placed along an imaginary half-circle appeared and children were asked, “Which one of these is the same like these two? [pointing to the objects along the imaginary half-circle].” The child was then allowed to touch one of the six items to indicate their choice. The task order was randomized.

At 45-months, children completed DL tasks for color and shape production and comprehension, in addition to the DCCS and Flanker (Fig. 2). In the DCCS, children were shown a test card and were instructed to sort to one of two locations marked by images of trays with target cards. During the pre- and post-switch phases children sorted yellow houses and purple fish to purple house and yellow fish target cards. Children indicated their choice by touching the sorting location on the screen. The experimenter first demonstrated sorting two trials by the pre-switch dimension to the children. This was followed by five pre-switch trials, five post-switch trials, and 30 mixed block trials in which the relevant dimension changed between trials. On mixed block trials, the features of the objects were changed to sorting red chairs and green rabbits to green chair and red rabbit target cards, and children were instructed to sort by the pre-switch dimension on 10/30 trials and by the post-switch dimension on 20/30 trials. Children were first told, “Sometimes we are going to play the shape game, and sometimes we are going to play the color game. In the shape game, rabbits go here, and chairs go there. In the color game, red ones go here, and green ones go there.” The computer played an audio cue of either shape or color depending on the relevant sorting rule, and the experimenter repeated the rule for the child (i.e. “Now we’re playing the color/shape game”). Children were given both rules again if they sorted a mixed block trial incorrectly. The pre-switch sorting dimension was counterbalanced between children.

In the Flanker task, children were told they would be feeding animals and were shown various animals facing right or left. To feed the animals, they used a serial response pad to indicate which direction the center animal was facing. There were three different trial types. In neutral trials, children were only shown a single animal on the screen. In congruent trials, there were four identical animals flanking the center animal (two on each side), and all of the animals were facing the same direction. In incongruent trials, the flanking animals were facing the opposite direction as the animal in the middle of the screen. Children completed six practice trials and 45 test trials (15 of each trial type). The proportion of correct responses, in addition to reaction time were analyzed.

#### NIRS recordings and analysis

Functional near-infrared spectroscopy (fNIRS) was used to monitor cortical activity through changes in oxygenated and deoxygenated hemoglobin while children completed these tasks^46^. Data were collected at 25 Hz using a Techen CW6 system with wavelengths of 830 nm and 690 nm. To measure neural activation in left frontal, left temporal-parietal, and right parietal regions, children wore a 52 cm fNIRS hat fitted with four sources and eight detectors, which combined to a total of ten 3-cm long channels (Fig. 1). Three channels were over left frontal, four were over left temporal-parietal, and three were placed over right parietal cortex. After placing the hat on each child’s head, a Polhemus Digitizing System was used to record the locations of the sources and detectors, as well as five anatomical landmarks on each participants’ head (vertex (CZ), right ear, left ear, nasion, and inion) onto an individual atlas in AtlasViewer^47^.

HomER2 software^48^ was used to subtract the mean baseline from the raw data and convert them into an optical density measure. Motion artifacts in fNIRS usually occur because of shifts between the sources and the scalp. Because young children are especially prone to movement at this age, this study opted to use a correction technique that had the potential to preserve more trials. In this study, the modified wavelet-filtering technique was used to correct motion across each channel successively^49^, using an IQR threshold of 0.5. The data were then band-pass filtered to preserve frequencies between 0.01 and 0.5 Hz. Data were next converted to concentration values using the modified Beer-Lambert Law and the known extinction coefficients of oxygenated and deoxygenated hemoglobin with differential and partial pathlength factor values of 6.0.

Volumetric timeseries data were constructed from these cleaned data following the procedure outlined by Forbes et al.^50^. We used a 30-month-old child atlas to register the fNIRS data. First, we created a light model using the spatial coordinates of source-detector positions. Using AtlasViewer, we performed photon migration simulations to create sensitivity profiles by estimating the path of light for each channel using parameters for absorption and scattering coefficients for the scalp, cerebrospinal fluid (CSF), gray, and white matter^51,52^. We created sensitivity profiles with Monte-Carlo simulations of 10,000,000 photons for each channel to determine its spatial sensitivity^53^. Sensitivity profiles for each channel were thresholded at 0.0001 and combined to create participant-specific masks that reflected the cortical volume of all recording NIRS channels. After, these masks were used to create a group mask which included voxels in which at least 75% of participants contributed data.

Image reconstruction in NeuroDOT^54^ integrates the simulated light model with the pre-processed channel-space data to generate volumetric timeseries data. Channel data originally collected at 25 Hz were down sampled to 10 Hz to lessen computational demands. Proper estimation of near-infrared light diffusion in biological tissue is an issue specific to optical imaging, as image reconstruction of the NIRS data is subject to rounding errors and may lead to an under-determined solution^55^. We therefore used the Tikhonov regularization method to create voxel-wise timeseries data for HbO and HbR^56–58^. General linear modeling was then used to estimate the amplitude of HbO and HbR for each condition and participant across the measured voxels. AFNI’s 3dMVM function was used to perform group analyses^59^. We thresholded effect masks with a voxel-wise threshold of *p* < 0.05. AFNI’s 3dClustSim function was used to correct these ANOVA for multiple comparisons. In order to avoid elevated false-positive results associated with use of the typical spatial autocorrelation function (ACF)^60^, we opted to follow a mixed approach proposed by Cox et al.^61^. In this approach, the ACF is estimated with a function that contains both Gaussian and monoexponential components, instead of the previous Gaussian-shaped assumption. We therefore used AFNI’s 3dFWHMx function for each ANOVA to estimate the ACF values. After, these values were included in 3dClustSim to control for family-wise error using an alpha threshold of 0.01 and a voxelwise threshold of *p* = 0.05. Average HbO and HbR values were then extracted for any clusters with significant interactions, and we used SPSS (IBM, version 25) for any follow-up tests. Greenhouse–Geisser corrections for sphericity violations were applied when necessary, and Bonferroni corrections were used for multiple comparisons.

To investigate the relationship between activation in the DL tasks and behavioral performance, *t*-tests were run using AFNI’s *3dttest*++ program. HbO and HbR were tested against zero for the DL tasks collapsed across task (combining color, shape, and embedded) and across dimension (combining production and comprehension), with each participant’s average behavioral score for task or dimension entered as a covariate. Cortical regions that had Hb values that covaried with participants’ behavioral scores were then identified using 3dClustSim to control for family-wise error using an alpha threshold of 0.01 and a voxelwise threshold of *p* = 0.05. HbO and HbR concentration values were extracted to visualize the association between Hb and performance.
